# Investigating the relationship between temporomandibular disorders and personality traits

**DOI:** 10.22514/jofph.2025.075

**Published:** 2025-12-12

**Authors:** Alessandro Marchesi, Rachele Buttironi, Andrea Sardella

**Affiliations:** ^1^Department of Gnathology, San Paolo Hospital, University of Milan, 20142 Milano, Italy

**Keywords:** Temporomandibular disorders, Personality traits, Facial pain

## Abstract

**Background**: This observational study investigates the potential link between temporomandibular disorders (TMD) and personality disorders. The Personality Inventory for Diagnostic and statistical manual of mental disorders (DSM)-5-Brief Form (PID-5-BF) was used to assess personality traits, while TMD diagnosis was established by combining dental and clinical history with specific screening questionnaires, including the TMD-Pain Screener. **Methods**: The study sample was recruited based on inclusion criteria requiring the presence of TMD-related symptoms (pain, joint clicking or functional limitations of the temporomandibular joint), with no active systemic or psychiatric conditions. Collected data were analyzed using descriptive and inferential statistical methods to identify significant correlations between PID-5-BF scores and the severity of temporomandibular symptoms. **Results**: Results highlight a relevant connection between specific personality domains and TMD symptom severity, suggesting that psychological factors may influence both the onset and persistence of the disorder. Statistically significant associations were found in the domains of antagonism (*p* = 0.039), negative affectivity (*p* = 0.024), and the mean total PID-5-BF score (*p* = 0.021), confirming the role of specific personality traits in modulating temporomandibular pain. **Conclusions**: These findings underscore the importance of an integrated approach, combining dental and psychological expertise, to improve clinical management and develop more effective prevention and treatment strategies.

## 1. Introduction

Temporomandibular disorders (TMD) are complex clinical conditions characterized by orofacial pain, joint dysfunction and muscular impairments, significantly impacting patients’ quality of life. It is one of the main causes of chronic non-odontogenic pain in the general population and can be associated with functional limitations, masticatory difficulties, and sleep disturbances, thus negatively influencing the psychophysical well-being of the individual [1]. Scientific literature has widely demonstrated the influence of psychological factors, such as stress and negative emotions, on pain perception in TMD [2, 3, 4]. Stressful life events, anxiety and depressive traits have been identified as triggers, aggravators, and perpetuators of symptoms, also affecting treatment responses [5, 6, 7]. These findings highlight the need for a multidimensional approach in evaluating and managing TMD. While the role of emotions in chronic pain modulation is well-established, few studies have specifically explored the correlation between TMD and personality disorder conditions marked by dysfunctional emotional regulation, affective instability and interpersonal difficulties [8]. Personality disorders are often associated with heightened emotional dysregulation, which may influence the central pain process. Emerging evidence suggests that individuals with dysfunctional personality traits may exhibit lower pain thresholds, increased sensitivity to nociceptive stimuli, and a tendency toward pain catastrophizing, potentially contributing to TMD symptom chronicity [9, 10, 11]. However, the relationship between personality traits and TMD remains understudied.

This study aims to investigate the association between dysfunctional personality traits and TMD symptoms, filling a gap in the current literature. Through a multidisciplinary analysis integrating dental and psychological assessments, this research explores the potential impact of personality traits on pain perception, symptom severity and clinical management. Identifying such correlations may offer new insights for targeted, personalized therapeutic approaches, ultimately improving the quality of life for TMD patients [12].

## 2. Materials and methods

The present study is an analytical observational case-control design conducted over 1.5 years between April 2023 and October 2024 at the Operative Unit of Odontostomatology of ASST Santi Paolo e Carlo, directed by Professor Andrea Sardella. The case group included symptomatic TMD patients recruited from the Gnathology department of the G. Vogel Dental Clinic on Via Beldiletto, while the control group comprised individuals not affiliated with this department. Both groups were administered the Personality Inventory for Diagnostic and statistical manual of mental disorders (DSM)-5-Brief Form (PID-5-BF) questionnaire to assess the prevalence of potential personality dysfunctions or specific personality domains. All subjects participating in the research signed a written or digital consent.

### 2.1 Inclusion and exclusion criteria

The case group included 134 patients (30 males, 104 females), aged 18–85 years diagnosed with temporomandibular disorders by combining dental and clinical history, and by using a gnathological pain screening questionnaire published by the International Network for Orofacial Pain and Related Disorders Methodology. According to literature [13], the positivity threshold for the long-form TMD Pain Screener is a score of 3, where response “a” assigns 0 points, “b” 1 point, and “c” 2 points. We didn’t distinguish individuals suffering from chronic pain disorder in the case group with TMD. Participants were required to have mastery in Italian. Exclusions included patients with cognitive deficits, neurodegenerative disorders, poor Italian mastery or a psychology degree.

The control group included 134 patients (30 males, 104 females), age 18–85 years. Controls completed the questionnaire either in waiting areas of other departments or digitally via social media, to ensure a representative sample of the general population. Age stratification (young adulthood: 18–39; middle adulthood: 40–69; advanced adulthood: 70+) reflected psychosocial developmental stages: career and relationship consolidation in young adulthood, generativity *vs.* stagnation in middle adulthood, and life reflection in advanced adulthood [14]. Age and sex matching in the case and control groups was insured by the association of same gender individuals and by the subdivision of different stages of life. Meanwhile age and gender stratification allowed to include the variations in pain modulation, which exhibit significant changes among individuals of different ages and genders [15]. Questionnaires with a raw score of 0 were excluded to avoid bias.

### 2.2 Personality inventory for DSM-5 BF (PID-5-BF)—adult

The PID-5-BF, administered voluntarily and anonymously to cases and controls, was developed by Krueger *et al*. [16] (2012) to create an empirically based diagnostic model. The hybrid DSM-5 Section III model integrates a dimensional approach with DSM-IV criteria [17]. The Diagnostic Criteria were split into two different ways: Criterion A analyzing impairments in self-functioning (identity, self-direction) and interpersonal functioning (empathy, intimacy), while Criterion B analyzing pathological personality traits organized into five domains: Negative Affectivity (intense negative emotions (*e.g.*, anxiety, guilt, anger), Detachment (avoidance of socioemotional experiences and reduced affective expression), Antagonism (exaggerated self-importance, lack of empathy and exploitative behavior), Disinhibition (impulsivity and disregard for consequences) and Psychoticism (culturally incongruent, bizarre thoughts or behaviors) [18]. Domains align with internalizing (negative affectivity, detachment) and externalizing (antagonism, disinhibition) spectra, reflecting common mental disorder comorbidities [18].

### 2.3 PID-5-BF analysis method

The PID-5-BF, a short-form screening tool, assesses five maladaptive trait domains (25 items total) via a 4-point Likert scale (0: Never or rarely false; 3: Always or often true). It does not provide a complete daignosis but identifies personality disorder in adolescents and adults. However, it does not evaluate Criterion A or include validity checks. The total score indicates the level of personality dysfunction and domain scores describe the traits [18]. The interpretation is based on *T*-scores normalized from the Italian manual of the PID-5-BF, which according to the Minnesota Multiphasic Personality Inventory does not require gender distinction. Scores of 60–65 indicate possible dysfunction, while values over 70 suggest a significant risk of personality disorder [19]. Each domain has a score from 0 to 15, elevated values reflect greater dysfunction in that trait. To process data, the clinicians examine each item’s score and record the raw values, calculate domain/total means, and compare them to normative data. This method proved reliable and clinically useful in DSM-5 Field Trials [16].

## 3. Results

The questionnaire scores were analyzed as previously described by a single examiner. After confirming compatibility with general population norms by comparing results to normalized *T*-scores, statistical analysis was performed (Table 1). The *p*-values indicate that the data does not follow a normal distribution, consistent with the nature of introspective analyses of individual emotions and perceptions, where conflicts between self-perception and social conformity may emerge. Comparison with standardized *T*-scores revealed no deviations from normative values, suggesting no significant personality disorder prevalence in the sample. However, differences between cases and controls were observed.

**Table 1.** Kolmogorov-Smirnov test for a single sample.

		Raw Total Score	Average Total Score	Negative Affectivity	Detachment	Antagonism	Disinhibition	Psychoticism
Sample, N		268	268	268	268	268	268	268
Normal Parameters								
	Media	20.550	0.820	1.170	0.780	0.620	0.772	0.780
	Standard Deviation	9.935	0.397	0.612	0.525	0.461	0.5642	0.559
Most Extreme Differences								
	Extreme	0.059	0.059	0.078	0.120	0.160	0.108	0.128
	Positive	0.059	0.059	0.068	0.120	0.160	0.108	0.128
	Negative	−0.035	−0.035	−0.078	−0.068	−0.090	−0.086	−0.081
Statistic Test		0.059	0.059	0.078	0.120	0.160	0.108	0.128
Asymtotic Significance (Two-Tailed)	Lilliefors significance correction	0.026	0.026	<0.001	<0.001	<0.001	<0.001	<0.001

We analyzed the Independent Samples Test in Table 2: a statistically significant difference was found between case and control groups for total raw score (*p*: 0.016), mean total score (*p*: 0.016), and the domains of negative affectivity (*p*: 0.017), detachment (*p*: 0.052), and antagonism (*p*: 0.012). No significant differences were observed for disinhibition or psychoticism.

**Table 2.** Independent samples test.

Equal variances assumed Equal variances not assumed	Levene’s Test for Equality of Variances		Test for Equality of Means						
	*F*	Sign.	*t*	*df* (degrees of freedom)	Significance (Two-Tailed)	Difference of the Mean	Standard Error Difference	95% Confidence Interval	
								Inferior	Superior
Raw Total Score	0.077	0.781	−2.426	266 264.614	0.016	−2.918	1.203	−5.286	−0.550
Average Total Score	0.077	0.781	−2.426	266 264.614	0.016	−0.117	0.048	−0.211	−0.022
Negative Affectivity	0.082	0.775	−2.398	266 265.133	0.017	−0.178	0.074	−0.323	−0.032
Detachment	0.890	0.346	−1.955	266 261.613	0.052	−0.125	0.064	−0.250	−0.001
Antagonism	7.828	0.006	−2.543	266 249.809	0.012	−0.142	0.056	−0.252	−0.032
Disinhibition	1.229	0.269	−1.040	266 257.621	0.299	−0.072	0.069	−0.207	−0.064
Psychoticism	0.046	0.830	−0.634	266 265.111	0.527	−0.043	0.068	−0.178	−0.091

The case group exhibited higher scores than the control group in total raw score, mean total score, negative affectivity, detachment and antagonism (Table 3).

**Table 3.** Group statistic.

		Number	Mean	Standard Deviation	Standard Error Mean
Raw Total Score					
	Controls	134	19.090	9.482	0.819
	Cases	134	22.010	10.195	0.881
Average Total Score					
	Controls	134	0.760	0.379	0.033
	Cases	134	0.880	0.408	0.035
Negative Affectivity					
	Controls	134	1.080	0.589	0.051
	Cases	134	1.260	0.623	0.054
Detachment					
	Controls	134	0.720	0.487	0.042
	Cases	134	0.840	0.555	0.048
Antagonism					
	Controls	134	0.550	0.394	0.034
	Cases	134	0.690	0.511	0.044
Disinhibition					
	Controls	134	0.736	0.511	0.044
	Cases	134	0.807	0.613	0.053
Psychoticism					
	Controls	134	0.760	0.543	0.047
	Cases	134	0.800	0.575	0.050

Table 4 shows the Non-Parametric Mann-Whitney Test. Consistent with prior results, the case group showed statistically significant differences in mean total score (*p*: 0.021), negative affectivity (*p*: 0.024), and antagonism (*p*: 0.039). Detachment differences, however, were marginally significant (*p* = 0.094), suggesting low-level significance.

**Table 4.** Non-parametric Mann-Whitney test.

	Null Hypothesis	Test	Sign	Decision
1	The distribution of the raw total score is the same across the group categories	Mann-Whitney U Test for Independent Samples	0.021	Reject the null hypothesis.
2	The distribution of the average total score is the same across the group categories	Mann-Whitney U Test for Independent Samples	0.021	Reject the null hypothesis.
3	The distribution of negative affectivity is the same across the group categories	Mann-Whitney U Test for Independent Samples	0.024	Reject the null hypothesis.
4	The distribution of detachment is the same across the group categories	Mann-Whitney U Test for Independent Samples	0.094	Retain the null hypothesis.
5	The distribution of antagonism is the same across the group categories	Mann-Whitney U Test for Independent Samples	0.039	Reject the null hypothesis.
6	The distribution of disinhibition is the same across the group categories	Mann-Whitney U Test for Independent Samples	0.686	Retain the null hypothesis.
7	The distribution of psychoticism is the same across the group categories	Mann-Whitney U Test for Independent Samples	0.657	Retain the null hypothesis.

Age- and sex-stratified analyses revealed no statistically significant differences.

## 4. Discussion

This study explored the correlation between temporomandibular disorders (TMD) and personality traits, focusing on psychological and somatic factors influencing symptom onset and chronicity. While *T*-scores aligned with normative data, deeper analysis revealed higher personality dysfunction in TMD patients, particularly in negative affectivity and antagonism. These traits marked by intense negative emotions, hostility and relational difficulties may perpetuate pain by amplifying emotional dysregulation. Emotional dysregulation is hypothesized to alter pain perception, contributing to TMD onset and chronicity [20, 21]. Negative affectivity and chronic stress may act as risk factors, especially in patients without global somatic symptoms [22, 23]. Emerging evidence suggests that unrecognized or mismanaged emotions exacerbate pain perception [24], highlighting the role of emotional regulation in TMD prevention and treatment [25, 26]. The results suggest that emotions influence pain perception in patients, independently of the presence of a personality disorder. However in these subjects, emotional dysregulation is intrinsic, so the removal of stressogenic factors might not be sufficient to alleviate symptomatology. Several studies support the importance of psychological aspects in pain management, highlighting the efficacy of Pain Neuroscience Education (PNE) in musculoskeletal pain management [27]. This educational approach uses metaphors and visuals to reframe pain neurobiology, reduce fear, and correct structural misconceptions [28, 29, 30]. Cognitive-Behavioral Therapy (CBT) has also proven effective in treating TMD; it teaches stress management and emotional regulation strategies, thus reducing pain impact and muscle activity in TMD [31]. In conclusion, the study highlights the relevance of psychological and personality factors in the treatment of TMD. Further research is necessary to clarify the role of negative emotions, stress and emotional dysregulation in the chronicization of symptoms, but current data suggest that integrating psychological approaches into therapeutic protocols could significantly improve clinical outcomes.

It is essential to consider the limitations of this study: a significant obstacle is the difficulty in completely isolating confounding variables (*e.g.*, sociodemographics, lifestyle, preexisting conditions). The use of a brief questionnaire, while pragmatic, might have limited some relevant details. Furthermore, emotional and psychological data are inherently influenced by social context, health, and life experiences, thereby complicating TMD correlation analyses. Larger samples and more detailed assessment tools could enhance validity and further clarify the complexity of the relationship between psychological factors and TMD.

## 5. Conclusions

These findings underscore the importance of integrating psychological and physical approaches in TMD management. Recognizing emotional components as key factors enables the inclusion of therapies like cognitive-behavoral therapy alongside traditional gnathological treatments, thereby improving symptom relief, stress management and emotional regulation. A holistic, multidisciplinary framework may optimize clinical outcomes and patient well-being and may be inspiration for future research on psychological factors in TMD chronicity.
